# Impact of changes in extracellular matrix in the lumbar degenerative disc

**Published:** 2011-08-25

**Authors:** G David, AV Ciurea, M Mitrica, A Mohan

**Affiliations:** * ‘Regina Maria’ Military Hospital, Neurosurgery Clinic, Brasov Romania; ** ‘Bagdasar Arseni’ Clinical Emergency Hospital, Neurosurgery Clinic, Bucharest Romania; *** ‘Carol Davila’ Central Military Clinical Hospital, Neurosurgery Clinic, BucharestRomania; **** Faculty of Medicine, Oradea Romania

**Keywords:** intervertebral disc degeneration (IDD), MMP 9 (metalloproteinase–9), TIMP–1 (tissue inhibitor of metalloproteinases–1), quality of life, extracellular matrix changes

## Abstract

The complexity of the clinical, biochemical, hystochemical and immunologic aspects of the intervertebral disk, along with its molecular biology, justifies the object of our study on the extracellular matrix modifications in lumbar disk hernias and their impact on patient quality of life.

Material and method: the research lot was composed of 50 patients, aged between 18 and 73, who have undergone lumbar disk hernia surgery. MMP–9 (metalloproteinase–9) and TIMP–1 (tissue inhibitor of matrix metalloprotease 1) have been dosed in order to study the modifications on extracellular disk matrix, and quality of life assessment was carried out both in pre–operatory and post–operatory periods.

Conclusions: patients may prevent the appearance of degenerative processes of the intervertebral disk with care and responsibility by controlling their weight, avoiding intense physical activities and ceasing to smoke.

## Introduction

Back pain is a major cause of discomfort and invalidity. This being said, the current treatment strategies are primarily focused on the improvement of symptoms and have different rates of success. For the future, and for the treatments to become pro–active, they have to turn towards cellular pathology. Intervertebral disk degeneration (IDD) has been linked to a large percentage of patients with lumbago, so inhibitory actions on the processes that contribute to IDD and the regeneration of the intervertebral disk matrix are a new current in research. Therapies aiming to inhibit cytokines like IL–1, that have a high level during IDD, have been investigated as possible treatments [1–5].

The degeneration of the disk is present during the natural process of aging, but there have been notes on accelerated degeneration processes in a series of young patients, underlining the pluri–factorial characteristic of this process. IDD is influenced by mechanical pressures, genetic inheritance and bio–cellular modifications [6–8].

Chondrocyte–like cells of the nucleus pulpous are responsible for the production of growth factors such as bone morphogenetic protein (BMP), transformal growth factor (TGF), insulin-like growth factor (IGF) and cytokines such as IL–1, IL–6 and tumor necrosis factor (TNF) [8]. Growth factors are implicated in the regulating of the intervertebral cellular matrix by stimulating the production of matrix proteins. In contrast, cytokines are inhibitors of matrix synthesis and stimulate the production of degrading enzymes like MMPs that brake the extracellular matrix of the intervertebral disk [6,9–12]. Chondrocyte–like cells are implicated in matrix synthesis, but they also produce enzymes implicated in the intervertebral degeneration process. Chondrocyte–like cells have also been identified in anomalies of the homeostasis process of the nucleus pulpous.

The increase in the level of MMP is a characteristic of ODD, especially considering MMP–17, 9 and 13, which mediate the degrading of matrix collagen type II [13–15]. MMP enzymes are produced in a latent form and impose this, in order to permit their proteolysis activation. Furthermore, the tissue inhibitor of the matrix metalloprotease (TIMP), which is found in four active forms – TIMP 1, 2, 3 and 4 blocks the activity of MMP. Types 1 and 2 are the most important MMP inhibitors. MMPs like MMP–7 have been found to be resistant to TIMP 1 and 2, so they have been noted as key enzymes in the pathogenesis of IDD [16–19].

The composition of the matrix and the activity of the intervertebral disk are integrated parts of its functionality. IDD leads to loss of disk matrix material, loss of chondrocyte–like cells of the nucleus pulpous – which leads to weaknesses in the structural integrity, hydration modifications, decrease of mechanical capacity, angiogenesis and innervations modifications. These biological changes resulting in IDD, associated with the decrease of disk height, produce pain and, implicitly, alter the quality of the patient's life [15,20,21].

## Methods

We have made a longitudinal, analytical, cohort study with an ambispective characteristic through which we can underline the histo–bio–chemical aspects of IDD pathology, correlated with the clinical aspects, risk factors, pre–surgery investigations and patient quality of life before and after the surgical intervention. The target population of this paper is represented by patients who had surgery for disk herniation during 01.01.2006 and 30.06.2009, in the neurosurgery clinic of ‘Regina Maria’ Military Emergency Hospital in Brasov. We have studied the impact of the IDD process (modifications in the extracellular matrix) on the pre and post–surgery patient's quality of life, alteration of health parameters (pain, mobility, usual activities, depression and self–care) on 50 patients that were eligible for prevailing intervertebral disk fragments.

Lumbar disk hernia diagnosis was made on anamnesis, neurological exam and neuro–imagistic imaging. For anamnesis, the patients were inquired according to the hospital chart and the Euro Quality of Life 5D (EQ–5D). This survey is a standardized instrument for the evaluation of quality of life. It consists of a self–assessment scale applied on large–scale in different clinical and treatment situation aiming to determine the quality of life according to 5 basic criteria–mobility, self–care, usual activities, pain/discomfort, and unease/depression. The scale has 2 parts. In the first part, each criterion has 3 expression grades. The patient checks the grade that corresponds to his state. The establishment of the quotient is made by using the EQ–5D index. The 2nd part represents an analogical–visual scale with a length of 20 centimeters, graded 0 to 100 where 0 represents the worst imaginable state of health and 100 the best. The 2nd part scale is also known as the ‘thermometer of health–state’. Appreciating to what degree the quality of life was affected, was made using both instruments. The evaluation was made both pre–surgery and 3 months post–surgery.

Disk materials were acquired during the surgical act and were fixed in parfine. The histo–pathologic study on the 50 selected patients was made by using usual coloration (Hematoxiline–Eosin) and special colorations (PAS, Giemsa and Van Gieson). For emphasizing on extracellular matrix modifications, we have studied:

- MMP–9 (metalloproteinase–9, gelatinase B or collagenase IV with a molecular mass of 92 kDa), a proteolytic enzyme implicated in the degrading conjunctive tissue. MMPs have a common path of activation, conservation of the amino acid sequence at the active link site and are inhibited by TIMPs. MMPs play an important role in angiogenesis control and in tumor cell invasion (and are used as predictive factors for this);
- TIMP–1, that plays an important role in the control of MMPs activity. TIMPs constitute a family of at least 3 types of proteins of which TIMP–1 and TIMP–2 are shown in a large variety of tissue types. TIMP–1 and TIMP–2 are inhibitors (in variable proportions) of all types of active MMPs. They bind with proMMP–9 and proMMP–2 and form stable complexes that promote the activation of pro-enzymes.

## Results

For MMP–9 we have observed positive results of different intensities at extracellular matrix level in 34 subjects (68%), in 16 cases (32%) the results were negative. (Table 1)

**Table 1:** Results for MMP–9

MMP–9 Reaction	n	Percent
Negative	16	32.0%
Positive for chondrocyte like	4	8.0%
Positive for chondrocyte like and matrix	11	22.0%
Positive for chondrocyte like and weak for matrix	2	4.0%
Positive for matrix	11	22.0%
Weak for matrix and chondrocyte like	6	12.0%
Total	50	100.0%

**Figure 1:**
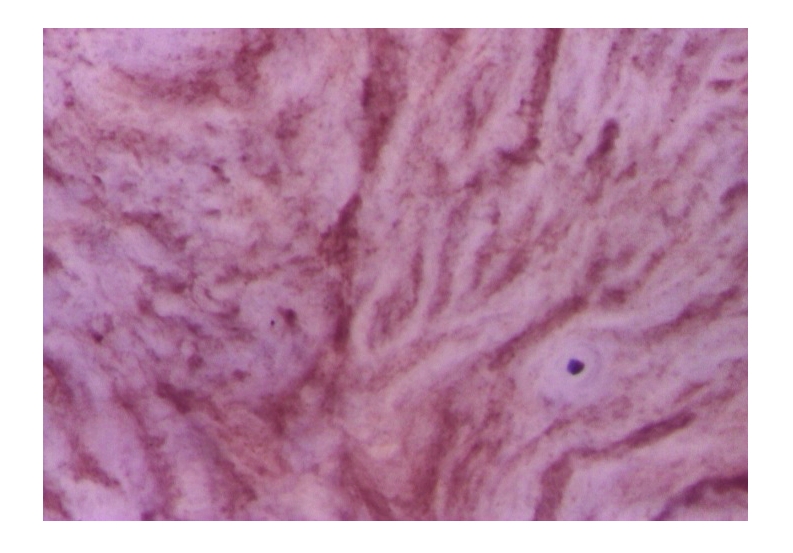
Intervertebral disk TIMP–1 positive in the extracellular matrix IHC 200X

The results of the research for TIMP–1, in the study, shows a positive reaction in all subjects, but of different intensity at the level of the cellular matrix. (Figure 1)

The analysis of the data shows that the modification of the extracellular matrix of the disk does not influence the intensity of the pain in disk hernia. There is no association between the hyper–algic form of hernia and the histo–pathological modifications of the MMP–9 positive type (95% confidence level X^2^=0,14)

The evaluation of the health thermometry in post–surgery time shows a lack of epidemiologic association between the extracellular modification, and an unsatisfactory improvement in the quality of life, from the point of view of health thermometry. However, for the clinician, this is important: only 70.5% of the MMP–9 positive subjects have reached a score over 70 post–surgery, in comparison with 81.25% of the MMP–9 negative subjects. The risk of those with positive MMP–9 histo–pathologic results, not very satisfying in improving their quality of life is 1.75 higher than in those without extracellular matrix modifications. [X^2^=0,64; RR=1,57 (0,5<RR<4,94)]

Although there is no association between the hyper–algic form of hernia and histo–pathological modification of the MMP–9 positive type, following a more profound analysis of many health state parameters, our study shows that the average EQ–5D pre–urgery score of the MMP–9 positive subjects is 0.4969, in comparison to 0.5623 for the MMP–9 negative ones (X^2^=9), therefore, we can establish that the modification in the extracellular disk matrix, significantly affects the patient's pre–surgery quality of life, both statistically and clinically (Table 2).

**Table 2:** Frequency of MMP–9 reactions and average EQ – 5D

MMP 9	n	Total	Average EQ–5D
negative	16	8.9972	0.5623
pos chondrocyte like	4	2.3119	0.5780
pos chondrocyte like and matrix	11	4.8445	0.4404
pos chondrocyte like and weak matrix	2	1.1583	0.5791
pos matrix	11	5.9869	0.5443
weak pos matrix and chondrocyte like	46	2.5939	0.4323

The risk for depression on those with MMP–9 positive is 1.58 times higher than those with negative results, with statistical significance [X^2^=6,49. (1<RR<2,56).RA=0,31]; 88,2% of the patients with positive MMP–9 have related episodes of depression in pre–operatory stage versus 56.2% of those without extracellular disk matrix modifications.

The risk of not improving their state of unease or depression after 3 months of the surgery on those with positive MMP–9, is statistically significant, higher than those with negative MMP–9 results (X^2^=5,16, RA=0,26). Clinically, we observed that the modification of the extracellular disk matrix has a negative impact on the improvement of the post–surgery psychical state, only 73,5 of those with positive MMP–9 have declared the lack of pre–operatory depression, in comparison with the 100% MMP–9 negative subjects.

Our study shows a lack of correlation between smoking and the modification of extracellular disk matrix. The risk of presenting MMP–9 positive results on smokers is 1.4 times higher than in non-smokers [RR= 1,4 (1<RR<1,96), for a 95%CI X^2^=2,6, , RA=0,23]. The prevalence of extracellular disk matrix modification in smokers is higher than in non–smokers (85,71% versus 61,11%)

The evaluation of obesity shows a lack of epidemiological association between excess weight and extracellular modification of the intervertebral disk. The risk to present MMP–9 positive results in obese or over–weight persons is 1.12 times higher than in normal weighted persons. The effect, although of no statistical importance, has a high clinical impact [RR= 1,12 (0,76<RR<1,66) for a CI of 95%, X^2^=0,34, , RA=0,07]. The prevalence of extracellular disk matrix modification in those with pathological body mass index is higher than in normally–weighted persons (71,4% versus 63,63%).

Our studies show a lack of association between the sedentary way of life and extracellular modification of the intervertebral disk. The risk to present MMP–9 positive results in sedentary subjects by nature of occupation and way of life is 1.14 higher than in those who have an activity corresponding to a good physical shape. [RR= 1,14 (0,71<RR<1,84) For a CI of 95%, X^2^=1,14, RA=0,08]. The studied effect of the obtained differences, although of no statistical importance, has a major clinical importance. The prevalence of extracellular disk modifications in sedentary subjects is higher than in those with a healthy lifestyle (70,2% versus 61,5%).

The analysis of our data shows a lack of association between the effort risk factor and the process of extracellular disk matrix modification. The risk to present positive MMP–9 results in those who overwork their back muscles is 1.11 higher than in those who do no cross the boundaries of maximal biomechanical stress of back muscles [RR= 1,11 (0,76<R<1,63) for a CI of 95%, X^2^=0,34, RA=0,07]. The prevalence of extracellular disk modifications in those who overwork the back from a biomechanical point of view is higher than in those without excessive efforts (70,2% versus 61,5%).

**Figure 2:**
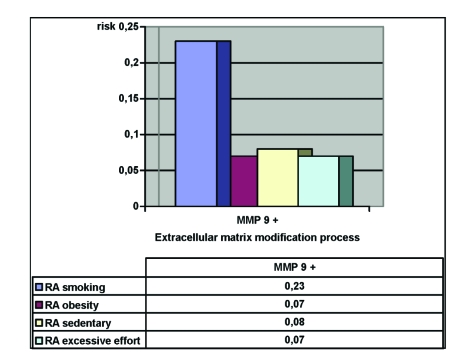
Risk that may be attributed to the conduct, that can be avoided and influence the extracellular matrix modification in IDD

The risk attributed to the relative risk characterizes both the aggressiveness of the risk factor and the frequency of the mentioned affection, so that it serves for the identifying of those issues that can be addressed and solved with maximum efficiency, and an optimal use of available resources. By eliminating smoking, we would reduce the extracellular matrix modifications by 23%. Having an adequate control of the weight, we would eliminate 7% of extracellular matrix modifications. Doing therapy and exercise aiming to harden the abdominal muscles, as well as the para–vertebral muscles, would eliminate 8% of these processes while ceasing excessive efforts would eliminate 7%. (Figure 2) 

## Discussions

The decrease in the number of notochord cells of the nucleus pulpous is associated with senescence and IDD (increased collagen synthesis, decreased secretion of proteoglicans and decreased water content). The reducing of the number of notochord cells after birth may be explained by their death and replacement with chondrocytes or chondrocytary differentiation [14].

Biochemical studies made on degenerated disks have shown increased catabolic enzyme levels and MMP, suggesting their role in degenerative processes. Gene therapy with anabolic factors for degenerative modifications of the intervertebral disk leads to increased proteoglican synthesis (predominantly aggrecan) and type II collagen.

Proteoglicans are synthesized by notochord cells from the nucleus pulpous. In degenerative processes, there is a modification in the synthesis of matrix molecules with increased synthesis of collagen types I and III and the decreased production of proteoglicans. Using TIMP–1 as an MMP inhibitor in gene therapy may become a complementary method for solving the degenerative process at disk level. In the catabolic process of IDD, MMP levels increase and TIMP levels decreased under the control of growth factors and cytokines (TNF alpha and IL–1). Studies made at disk level have emphasized the rise in MMP–1, –9 and –13. The expression of TIMP–1 and –2 was increased in degenerated disks, while TIMP–3 expression was not significantly high in the degeneration process. These results suggest that high expression of MMP is countered by the high expression of TIMP [19].

In this study, we have concluded that high MMP–9 expression is sometimes correlated with high TIMP–1 expression.

Another aim of our research was to determine the impact of risk factors on the cellular biology of the degenerated intervertebral disk. In disk hernia we have risk factors that cannot be avoided, such as aging, make gender, a history of lumbar trauma or surgery. The aging process of inferior lumbar intervertebral disks, as well as frequent disk or spinal muscle injury rise the predisposition for back pain, a process which debuts in adulthood and is frequently associated with depression and anxiety as a current unspecific method for coping with the disease state. Psychical dysfunctions per–se, as well as the psychical coloring of disk hernias, must be correctly assessed and inserted in the therapeutic conclusion [22].

Another category of factors is that made out of avoidable ones: work or other activities that rise the risk of disk hernia – like sitting for a long period of time, lifting heavy objects, frequent twists or bends, hard physical exercise, repetitive movements or constant vibrations, such as driving a car, incorrect movements and exercises, high tension in the back muscles, hard exercises after a period of inactivity, smoking or obesity. Nicotine and other toxins may deteriorate the capacity of the intervertebral disks to absorb nutrients from the blood, raising the possibility of degeneration or destruction, and the increase in body weight (especially abdominal weight) add to a high workload for the lumbar disks [23].

Some patients may prevent the apparition of degenerative processes of the intervertebral disk, or the aggravation of symptoms, if they are caring and responsible while maintaining their weight under control, adopting a correct posture, avoiding intense physical activities, ceasing to smoke and avoiding aggravating factors (damp, cold) [23].

## Conclusions

1. In this study, we have concluded that the presence of extracellular matrix modifications in lumbar disks and the increased expression of MMP–9 is correlated with the increase in TIMP–1 expression. (MMP–9 positive in 68% of subjects and 100% TIMP–1 positive). The high incidence of MMP positive results suggests their role in the degenerative process.
2. The modification of extracellular disk matrix has a significant negative statistic a and clinical impact on the improvement of post–surgery psychical state; this process being associated in epidemiology with a high recurrence of pre–surgery depression and a poorer pre–surgery quality of life (average EQ–5D pre–operatory in MMP–9 positive is 0,4969 versus 0,5623 in MMP–9 negative subjects. In chronic disk hernia, in moderate but repetitive crisis, the occurrence of a psychical reaction is justified. Presenting all these psychological coping mechanism in impact with an organic illness emphasizes a classical issue: the results of the surgical treatment of lumbar disk hernia are highly influenced by the psychopathologic element.
3. Smoking, excess weight, sedentary way of life and effort, raise the reoccurrence of extracellular disk matrix modification processes. By eliminating smoking with an adequate control of weight, by exercises aiming to strengthen the abdominal and the para–vertebral muscles, and by eliminating excessive efforts, we would reduce the extracellular disk matrix modification processes in the degenerative disk by 7% to 23%.
